# Fractional Hermite-Hadamard inequalities containing generalized Mittag-Leffler function

**DOI:** 10.1186/s13660-017-1543-4

**Published:** 2017-10-25

**Authors:** Marcela V Mihai, Muhammad Uzair Awan, Muhammad Aslam Noor, Khalida Inayat Noor

**Affiliations:** 1 Department Scientific-Methodical Sessions, Romanian Mathematical Society-branch Bucharest, Academy Street no. 14, Bucharest, RO-010014 Romania; 2 Department of Mathematics, GC University, Faisalabad, Pakistan; 3 Department of Mathematics, COMSATS Institute of Information Technology, Park Road, Islamabad, Pakistan

**Keywords:** 26D15, 26A51, 26A33, 33E12, harmonic convex function, Hermite-Hadamard inequalities, Mittag-Leffler function

## Abstract

The objective of this paper is to establish some new refinements of fractional Hermite-Hadamard inequalities via a harmonically convex function with a kernel containing the generalized Mittag-Leffler function.

## Introduction

A function $f:I\to\mathbb{R}$ is said to be convex if

$$f\bigl((1-t)x+ty\bigr)\leq(1-t)f(x)+tf(y),\quad\forall x,y\in I,t\in[0,1]. $$

Convexity plays a pivotal role in different fields of pure and applied sciences. Another fact that makes it more attractive is its close relationship with theory of inequalities. In particular, integral inequalities have been obtained via convex functions. Inspired by the research work in this field, many authors introduced new extensions of classical convex functions; see, for example, [1–4] and the references therein. Recently, Işcan [3] introduced and investigated the notion of harmonically convex functions. These days the class of harmonically convex functions is receiving much attention by many researchers. For more details, see [3–9]. Hermite and Hadamard independently obtained an integral inequality that provides us a necessary and sufficient condition for a function to be convex. This famous result reads as follows.

Let $f:I\supseteq[a,b]\rightarrow\mathbb{R}$ be a convex function, then

1.1 $$ f \biggl(\frac{a+b}{2} \biggr)\leq\frac{1}{b-a} \int _{a}^{b} f(x)\,\mathrm{d}x\leq\frac{f(a)+f(b)}{2}. $$

For more details on Hermite-Hadamard-type inequalities, see [1, 2, 4, 10, 11]. Sarikaya et al. [12] developed a new generalization of Hermite-Hadamard-type inequality using fractional calculus approach. This opened a new venue of research in this field. Utilizing the concepts of fractional calculus, Işcan et al. [6] derived new refinements of fractional Hermite-Hadamard’s inequality via harmonically convex functions. The main motivation of this paper is to obtain new refinements of fractional Hermite-Hadamard-type inequalities via harmonically convex functions in connection with the generalized Mittag-Leffler function, which even generalizes the classical Riemann-Liouville fractional integral operators. We also discuss some particular cases.

## Preliminaries

In this section, we discuss some preliminary concepts and facts. Recently, Işcan [3] obtained several inequalities of Hermite-Hadamard type via harmonic convex functions.

The class of harmonic convex functions is defined as follows.

### Definition 2.1

([3])

Let $f:I\subseteq\mathbb{R}\setminus \{ 0 \} \rightarrow \mathbb{R}$, where *I* a real interval. The function *f* is said to be harmonic convex if

2.1 $$ f \biggl( \frac{xy}{tx+(1-t)y} \biggr) \leq tf(y)+(1-t)f(x) $$

for all $x,y\in I$ and $t\in{[}0,1]$. If (2.1) holds in the reversed sense, then *f* is said to be harmonic concave.

Işcan et al. [6] established new fractional estimates of Hermite-Hadamard-type inequalities via harmonic convex functions. For more details on Hermite-Hadamard inequalities involving fractional integrals; see Mihai [13, 14], Mihai et al. [15], Awan et al. [16], Kunt et al. [7], Sarikaya et al. [17], Nisan et al. [18], and references therein. Latif et al. [8] gave the following definition.

### Definition 2.2

A function $g:[a,b]\subseteq\mathbb{R}\setminus \lbrace0 \rbrace\to\mathbb{R}$ is said to be harmonically symmetric with respect to $\frac{2ab}{a+b}$ if

$$ g(x)=g \biggl( \frac{1}{\frac{1}{a}+\frac{1}{b}-\frac{1}{x}} \biggr) $$

for all $x\in[a,b]$.

For details and the definition of Riemann-Liouville fractional integrals, see [19, 20].

Salim et al. [21] have defined the generalized fractional integral operators containing Mittag-Leffler function:

### Definition 2.3

Let $\mu, \nu, k, l, \gamma>0$ and $\omega\in\mathbb{R} $. Then the generalized fractional integral operators containing the Mittag-Leffler functions $\varepsilon_{\mu, \nu, l, \omega, a^{+}}^{\gamma, \delta, k}$ and $\varepsilon_{\mu, \nu, l, \omega, b^{-}}^{\gamma, \delta, k}$ for a real-valued continuous function *f* are defined by

2.2 $$ \bigl( \varepsilon_{\mu, \nu, l, \omega, a^{+}}^{\gamma, \delta, k}f \bigr) (x)= \int_{a}^{x}(x-t)^{\nu-1}E_{\mu, \nu, l}^{\gamma, \delta , k} \bigl( \omega(x-t)^{\mu} \bigr) f(t)\,\mathrm{d}t $$

and

2.3 $$ \bigl( \varepsilon_{\mu, \nu, l, \omega, b^{-}}^{\gamma, \delta, k}f \bigr) (x)= \int_{x}^{b}(t-x)^{\nu-1}E_{\mu, \nu, l}^{\gamma, \delta , k} \bigl( \omega(t-x)^{\mu} \bigr) f(t)\,\mathrm{d}t, $$

respectively, where $E_{\mu, \nu, l}^{\gamma, \delta, k}$ is the generalized Mittag-Leffler function defined as

$$ E_{\mu, \nu, l}^{\gamma, \delta, k}(t)=\sum_{n=0}^{\infty} \frac{(\gamma )_{kn}}{\Gamma(\mu n+\nu)}\frac{t^{n}}{(\delta)_{ln}}, $$

and $(a)_{n}$ is the Pochhammer symbol defined as

$$(a)_{n}=a(a+1)\cdots(a+n-1),\quad(a)_{0}=1. $$

### Remark

If $k=l=1$ in (2.2), then the integral operator $( \varepsilon_{\mu, \nu, 1, \omega, a^{+}}^{\gamma, \delta, k}f )$ reduces to the integral operator $( \varepsilon_{\mu, \nu, l, \omega, a^{+}}^{\gamma, \delta, 1}f )$ containing the generalized Mittag-Leffler function $E_{\mu, \nu, 1}^{\gamma, \delta, 1}$ introduced by Srivastava and Tomovski [22]. If $k=l=1$ and $\delta=1$, then (2.2) reduces to the integral operator defined by Prabhaker [23] and containing the Mittag-Leffler function $E_{\mu, \nu}^{\gamma}$. For $\omega=0$ in (2.2), the integral operator $( \varepsilon_{\mu, \nu, l, \omega, a^{+}}^{\gamma, \delta, k}f )$ reduces to the Riemann-Liouville fractional integral operator [21].

In [21] the properties of the generalized integral operator and the generalized Mittag-Leffler function are studied. It is proved that $E_{\mu, \nu, l}^{\gamma, \delta, k} ( t )$ is absolutely convergent for all $t\in\mathbb{R}$ if $k< l+\mu$. Since

$$\bigl\vert E_{\mu, \nu, l}^{\gamma, \delta, k} ( t ) \bigr\vert \leq\sum _{n=0}^{\infty} \biggl\vert \frac{(\gamma)_{kn}}{\Gamma(\mu n+\nu )} \frac{t^{n}}{(\delta)_{ln}} \biggr\vert $$

with

$$\sum_{n=0}^{\infty} \biggl\vert \frac{(\gamma)_{kn}}{\Gamma(\mu n+\nu )}\frac{t^{n}}{(\delta)_{ln}} \biggr\vert =S, $$

we have

$$\bigl\vert E_{\mu, \nu, l}^{\gamma, \delta, k} ( t ) \bigr\vert \leq S. $$

## Results and discussions

In this section, we discuss our main results. We write $\Vert g \Vert _{\infty}=\sup_{t\in[a,b]} \vert g(t) \vert $ for a continuous function $g:[a,b]\to\mathbb{R}$.

### Lemma 3.1

*If* $g:[a,b]\subseteq\mathbb{R}\setminus \lbrace0 \rbrace\to \mathbb{R}$ *is integrable and harmonically symmetric with respect to* $\frac{2ab}{a+b}$, *then*

$$\begin{aligned} \bigl( \varepsilon_{\mu, \nu, l, \omega, (\frac{a+b}{2ab} )^{+}}^{\gamma, \delta, k}g\circ h \bigr) \biggl( \frac{1}{a} \biggr) &= \bigl( \varepsilon_{\mu, \nu, l, \omega, (\frac{a+b}{2ab} )^{-}}^{\gamma, \delta, k}g\circ h \bigr) \biggl( \frac{1}{b} \biggr) \\ &=\frac{1}{2} \biggl[ \bigl( \varepsilon_{\mu, \nu, l, \omega, (\frac {a+b}{2ab} )^{+}}^{\gamma, \delta, k}g \circ h \bigr) \biggl( \frac {1}{a} \biggr)+ \bigl( \varepsilon_{\mu, \nu, l, \omega, (\frac {a+b}{2ab} )^{-}}^{\gamma, \delta, k}g \circ h \bigr) \biggl( \frac {1}{b} \biggr) \biggr] , \end{aligned}$$

*where* $h(x)=\frac{1}{x}$, $x\in [\frac{1}{b}, \frac{1}{a} ] $.

### Proof

Since *g* is harmonically symmetric with respect to $\frac{2ab}{b-a}$, using Definition 2.2, we have $g (\frac{1}{t} ) =g ( \frac{1}{\frac{1}{a}+\frac{1}{b}-t} )$ for all $t\in [\frac {1}{b}, \frac{1}{a} ] $. Hence, in the following integral, setting $u=\frac{1}{a}+\frac{1}{b}-t$ and $\mathrm{d}u=-\mathrm{d}t$ gives

$$\begin{aligned} & \bigl( \varepsilon_{\mu, \nu, l, \omega, (\frac{a+b}{2ab} )^{+}}^{\gamma, \delta, k}g\circ h \bigr) \biggl( \frac{1}{a} \biggr) \\ &\quad = \int_{\frac{a+b}{2ab}}^{\frac{1}{a}} \biggl(\frac{1}{a}-t \biggr)^{\nu -1}E_{\mu, \nu, l}^{\gamma, \delta, k} \biggl( \omega \biggl( \frac {1}{a}-t \biggr) ^{\mu} \biggr)g \biggl(\frac{1}{t} \biggr)\,\mathrm{d}t \\ &\quad = \int_{\frac{a+b}{2ab}}^{\frac{1}{a}} \biggl(\frac{1}{a}-t \biggr)^{\nu -1}E_{\mu, \nu, l}^{\gamma, \delta, k} \biggl( \omega \biggl( \frac {1}{a}-t \biggr) ^{\mu} \biggr)g \biggl( \frac{1}{\frac{1}{a}+\frac {1}{b}-t} \biggr)\,\mathrm{d}t \\ &\quad = \int_{\frac{1}{b}}^{\frac{a+b}{2ab}} \biggl(u- \frac{1}{b} \biggr)^{\nu -1}E_{\mu, \nu, l}^{\gamma, \delta, k} \biggl( \omega \biggl(u- \frac {1}{b} \biggr) ^{\mu} \biggr)g \biggl( \frac{1}{u} \biggr)\,\mathrm{d}u= \bigl( \varepsilon_{\mu, \nu, l, \omega, (\frac{a+b}{2ab} )^{-}}^{\gamma, \delta, k}g\circ h \bigr) \biggl( \frac{1}{b} \biggr) . \end{aligned}$$

This completes the proof. □

### Lemma 3.2

*Let* $f:I\subset(0, +\infty)\to\mathbb{R}$ *be a differentiable function on* $I^{\circ}$, *the interior of* *I*, *such that* $f^{\prime}\in L[a,b]$, *where* $a,b \in I$. *If* $g:[a,b]\to\mathbb{R}$ *is integrable and harmonically symmetric with respect to* $\frac{2ab}{a+b}$, *then the following equality holds*:

3.1 $$\begin{aligned} &f \biggl(\frac{2ab}{a+b} \biggr) \biggl[ \bigl( \varepsilon_{\mu, \nu, l, \omega^{\prime}, (\frac{a+b}{2ab} )^{+}}^{\gamma, \delta, k}g\circ h \bigr) \biggl( \frac{1}{a} \biggr)+ \bigl( \varepsilon_{\mu, \nu , l, \omega^{\prime}, (\frac{a+b}{2ab} )^{-}}^{\gamma, \delta, k}g\circ h \bigr) \biggl( \frac{1}{b} \biggr) \biggr] \\ &\qquad{}- \biggl[ \bigl( \varepsilon_{\mu, \nu, l, \omega^{\prime}, (\frac{a+b}{2ab} )^{+}}^{\gamma, \delta, k}fg\circ h \bigr) \biggl( \frac{1}{a} \biggr)+ \bigl( \varepsilon_{\mu, \nu, l, \omega^{\prime}, (\frac{a+b}{2ab} )^{-}}^{\gamma, \delta, k}fg \circ h \bigr) \biggl( \frac{1}{b} \biggr) \biggr] \\ &\quad = \int_{\frac{1}{b}}^{\frac{a+b}{2ab}} \biggl( \int_{\frac{1}{b}}^{t} \biggl(s-\frac{1}{b} \biggr) ^{\nu-1}E_{\mu, \nu, l}^{\gamma, \delta, k} \biggl( \omega^{\prime} \biggl(s- \frac{1}{b} \biggr) ^{\mu} \biggr) (g\circ h ) (s) \,\mathrm{d}s \biggr) (f\circ h ) ^{\prime }(t)\,\mathrm{d}t \\ &\qquad{}- \int_{\frac{a+b}{2ab}}^{\frac{1}{a}} \biggl( \int_{t}^{\frac {1}{a}} \biggl(\frac{1}{a}-s \biggr) ^{\nu-1}E_{\mu, \nu, l}^{\gamma, \delta, k} \biggl( \omega^{\prime} \biggl(\frac{1}{a}-s \biggr) ^{\mu} \biggr) (g\circ h ) (s) \,\mathrm{d}s \biggr) (f\circ h ) ^{\prime}(t)\,\mathrm{d}t, \end{aligned}$$

*where* $h(x)=\frac{1}{x}$, $x\in [\frac{1}{b}, \frac{1}{a} ]$, *and* $\omega^{\prime}= ( \frac{2ab}{b-a} ) ^{\mu}\omega$.

### Proof

It suffices to show that

3.2 $$\begin{aligned} I&= \int_{\frac{1}{b}}^{\frac{a+b}{2ab}} \biggl( \int_{\frac{1}{b}}^{t} \biggl(s-\frac{1}{b} \biggr) ^{\nu-1}E_{\mu, \nu, l}^{\gamma, \delta, k} \biggl( \omega^{\prime} \biggl(s- \frac{1}{b} \biggr) ^{\mu} \biggr) (g\circ h ) (s) \,\mathrm{d}s \biggr) (f\circ h ) ^{\prime }(t)\,\mathrm{d}t \\ &\quad{}- \int_{\frac{a+b}{2ab}}^{\frac{1}{a}} \biggl( \int_{t}^{\frac {1}{a}} \biggl(\frac{1}{a}-s \biggr) ^{\nu-1}E_{\mu, \nu, l}^{\gamma, \delta, k} \biggl( \omega^{\prime} \biggl(\frac{1}{a}-s \biggr) ^{\mu} \biggr) (g\circ h ) (s) \,\mathrm{d}s \biggr) (f\circ h ) ^{\prime}(t)\,\mathrm{d}t \\ &=I_{1}-I_{2}. \end{aligned}$$

By Lemma 3.1, integrating by parts, we have

3.3 $$\begin{aligned} I_{1}&= \int_{\frac{1}{b}}^{t} \biggl(s-\frac{1}{b} \biggr) ^{\nu -1}E_{\mu, \nu, l}^{\gamma, \delta, k} \biggl( \omega^{\prime} \biggl(s- \frac{1}{b} \biggr) ^{\mu} \biggr) (g\circ h ) (s) \,\mathrm{d}s \cdot (f\circ h ) (t) \bigg\vert _{\frac{1}{b}}^{\frac {a+b}{2ab}} \\ &\quad{}- \int_{\frac{1}{b}}^{\frac{a+b}{2ab}} \biggl(t-\frac{1}{b} \biggr) ^{\nu-1}E_{\mu, \nu, l}^{\gamma, \delta, k} \biggl( \omega^{\prime} \biggl(t- \frac{1}{b} \biggr) ^{\mu} \biggr) (g\circ h ) (t) (f \circ h ) (t)\,\mathrm{d}t \\ &=f \biggl(\frac{2ab}{a+b} \biggr) \int_{\frac{1}{b}}^{\frac {2ab}{a+b}} \biggl(s-\frac{1}{b} \biggr) ^{\nu-1}E_{\mu, \nu, l}^{\gamma, \delta, k} \biggl( \omega^{\prime} \biggl(s- \frac{1}{b} \biggr) ^{\mu} \biggr) (g\circ h ) (s) \,\mathrm{d}s \\ &\quad{}- \int_{\frac{1}{b}}^{\frac{a+b}{2ab}} \biggl(t-\frac{1}{b} \biggr) ^{\nu-1}E_{\mu, \nu, l}^{\gamma, \delta, k} \biggl( \omega^{\prime} \biggl(t- \frac{1}{b} \biggr) ^{\mu} \biggr) (fg\circ h ) (t) \,\mathrm{d}t. \end{aligned}$$

Thus

3.4 $$\begin{aligned} I_{1}&=f \biggl(\frac{2ab}{a+b} \biggr) \bigl( \varepsilon_{\mu, \nu, l, \omega^{\prime}, (\frac{a+b}{2ab} )^{-}}^{\gamma, \delta, k}g\circ h \bigr) \biggl( \frac{1}{b} \biggr)- \bigl( \varepsilon_{\mu, \nu , l, \omega^{\prime}, (\frac{a+b}{2ab} )^{-}}^{\gamma, \delta, k}fg\circ h \bigr) \biggl( \frac{1}{b} \biggr) \\ &=\frac{1}{2}f \biggl(\frac{2ab}{a+b} \biggr) \biggl[ \bigl( \varepsilon_{\mu , \nu, l, \omega^{\prime}, (\frac{a+b}{2ab} )^{+}}^{\gamma, \delta, k}g\circ h \bigr) \biggl( \frac{1}{a} \biggr)+ \bigl( \varepsilon _{\mu, \nu, l, \omega^{\prime}, (\frac{a+b}{2ab} )^{-}}^{\gamma, \delta, k}g \circ h \bigr) \biggl( \frac{1}{b} \biggr) \biggr] \\ &\quad{}- \bigl( \varepsilon_{\mu, \nu, l, \omega^{\prime}, (\frac {a+b}{2ab} )^{-}}^{\gamma, \delta, k}fg\circ h \bigr) \biggl( \frac {1}{b} \biggr). \end{aligned}$$

Analogously,

3.5 $$\begin{aligned} I_{2}&= \int_{t}^{\frac{1}{a}} \biggl(\frac{1}{a}-s \biggr) ^{\nu -1}E_{\mu, \nu, l}^{\gamma, \delta, k} \biggl( \omega^{\prime} \biggl(\frac {1}{a}-s \biggr) ^{\mu} \biggr) (g\circ h ) (s) \,\mathrm{d}s \cdot (f\circ h ) (t) \bigg\vert _{\frac{a+b}{2ab}}^{\frac {1}{a}} \\ &\quad{}- \int_{\frac{a+b}{2ab}}^{\frac{1}{a}} \biggl(\frac{1}{a}-t \biggr) ^{\nu-1}E_{\mu, \nu, l}^{\gamma, \delta, k} \biggl( \omega^{\prime} \biggl(\frac{1}{a}-t \biggr) ^{\mu} \biggr) (g\circ h ) (t) (f \circ h ) (t)\,\mathrm{d}t \\ &=-f \biggl(\frac{2ab}{a+b} \biggr) \int_{\frac{a+b}{2ab}}^{\frac{1}{a}} \biggl(\frac{1}{a}-s \biggr) ^{\nu-1}E_{\mu, \nu, l}^{\gamma, \delta, k} \biggl( \omega^{\prime} \biggl(\frac{1}{a}-s \biggr) ^{\mu} \biggr) (g\circ h ) (s) \,\mathrm{d}s \\ &\quad{}+ \int_{\frac{a+b}{2ab}}^{\frac{1}{a}} \biggl(\frac{1}{a}-t \biggr) ^{\nu-1}E_{\mu, \nu, l}^{\gamma, \delta, k} \biggl( \omega^{\prime} \biggl(\frac{1}{a}-t \biggr) ^{\mu} \biggr) (fg\circ h ) (t) \,\mathrm{d}t. \end{aligned}$$

Also,

3.6 $$\begin{aligned} I_{2}&=-f \biggl(\frac{2ab}{a+b} \biggr) \bigl( \varepsilon_{\mu, \nu, l, \omega^{\prime}, (\frac{a+b}{2ab} )^{+}}^{\gamma, \delta, k}g\circ h \bigr) \biggl( \frac{1}{a} \biggr)+ \bigl( \varepsilon_{\mu, \nu , l, \omega^{\prime}, (\frac{a+b}{2ab} )^{+}}^{\gamma, \delta, k}fg\circ h \bigr) \biggl( \frac{1}{a} \biggr) \\ &=-\frac{1}{2}f \biggl(\frac{2ab}{a+b} \biggr) \biggl[ \bigl( \varepsilon _{\mu, \nu, l, \omega^{\prime}, (\frac{a+b}{2ab} )^{+}}^{\gamma, \delta, k}g\circ h \bigr) \biggl( \frac{1}{a} \biggr)+ \bigl( \varepsilon _{\mu, \nu, l, \omega^{\prime}, (\frac{a+b}{2ab} )^{-}}^{\gamma, \delta, k}g \circ h \bigr) \biggl( \frac{1}{b} \biggr) \biggr] \\ &\quad{}+ \bigl( \varepsilon_{\mu, \nu, l, \omega^{\prime}, (\frac {a+b}{2ab} )^{+}}^{\gamma, \delta, k}fg\circ h \bigr) \biggl( \frac {1}{a} \biggr). \end{aligned}$$

Inserting (3.4) and (3.6) into (3.2), we get (3.1), and the proof is complete. □

### Remark

Taking $\omega=0$ in Lemmas 3.1 and 3.2, we have Lemmas 2 and 3 from [7].

The next result is an Hermite-Hadamard-type inequality for a generalized fractional integral operator containing the generalized Mittag-Leffler function.

### Theorem 3.3

*Let* $f:I\subset(0, +\infty)\to\mathbb{R}$ *be a function such that* $f\in L[a,b]$, *where* $a,b \in I$. *If* *f* *is a harmonically convex function on* $[a,b]$, *then the following inequality for fractional integrals holds*:

3.7 $$ \begin{aligned}[b]&f \biggl(\frac{2ab}{a+b} \biggr) \bigl( \varepsilon_{\mu, \nu, l, \omega ^{\prime}, (\frac{a+b}{2ab} )^{-}}^{\gamma, \delta, k}1 \bigr) \biggl(\frac{1}{b} \biggr) \\ &\quad \leq\frac{1}{2} \biggl[ \bigl( \varepsilon_{\mu, \nu, l, \omega^{\prime}, (\frac{a+b}{2ab} )^{-}}^{\gamma, \delta, k}f \circ h \bigr) \biggl(\frac{1}{b} \biggr)+ \bigl( \varepsilon_{\mu, \nu, l, \omega^{\prime}, (\frac{a+b}{2ab} )^{+}}^{\gamma, \delta, k}f \circ h \bigr) \biggl(\frac{1}{a} \biggr) \biggr] \\ &\quad \leq\frac{f(a)+f(b)}{2} \bigl( \varepsilon_{\mu, \nu, l, \omega^{\prime }, (\frac{a+b}{2ab} )^{+}}^{\gamma, \delta, k}1 \bigr) \biggl(\frac {1}{a} \biggr), \end{aligned} $$

*where* $h(x)=\frac{1}{x}$, $x\in [\frac{1}{b}, \frac{1}{a} ]$, *and* $\omega^{\prime}= ( \frac{2ab}{b-a} ) ^{\mu}\omega$.

### Proof

Since *f* is a harmonically convex function on $[a,b]$, we have

3.8 $$ f \biggl(\frac{2xy}{x+y} \biggr) \leq\frac{f(x)+f(y)}{2} $$

for $x,y\in[a,b]$. Substituting $x=\frac{ab}{\frac{t}{2}a+\frac{2-t}{2}b}$ and $y=\frac {ab}{\frac{t}{2}b+\frac{2-t}{2}a} $ into inequality (3.8), we get

3.9 $$ f \biggl(\frac{2ab}{a+b} \biggr) \leq\frac{f (\frac{ab}{\frac {t}{2}a+\frac{2-t}{2}b} ) +f (\frac{ab}{\frac{t}{2}b+\frac {2-t}{2}a} ) }{2}. $$

Multiplying both sides of (3.9) by $t^{\nu-1}E_{\mu, \nu, l}^{\gamma, \delta, k} ( \omega t^{\mu} )$ and integrating over $[0,1]$, we get

$$\begin{aligned} &2f \biggl(\frac{2ab}{a+b} \biggr) \int_{0}^{1}t^{\nu-1}E_{\mu, \nu, l}^{\gamma, \delta, k} \bigl( \omega t^{\mu} \bigr)\,\mathrm{d}t \\ &\quad \leq \int_{0}^{1}t^{\nu-1}E_{\mu, \nu, l}^{\gamma, \delta, k} \bigl( \omega t^{\mu} \bigr)f \biggl(\frac{ab}{\frac{t}{2}a+\frac {2-t}{2}b} \biggr) \,\mathrm{d}t+ \int_{0}^{1}t^{\nu-1}E_{\mu, \nu, l}^{\gamma, \delta, k} \bigl( \omega t^{\mu} \bigr)f \biggl(\frac {ab}{\frac{t}{2}b+\frac{2-t}{2}a} \biggr) \,\mathrm{d}t . \end{aligned}$$

Setting

3.10 $$ u=\frac{1}{ab} \biggl(\frac{t}{2}a+ \frac{2-t}{2}b \biggr),\qquad v=\frac {1}{ab} \biggl(\frac{t}{2}b+ \frac{2-t}{2}a \biggr), $$

we have

$$\begin{aligned} &2f \biggl(\frac{2ab}{a+b} \biggr) \int_{\frac{1}{b}}^{\frac {a+b}{2ab}} \biggl(v-\frac{1}{b} \biggr) ^{\nu-1}E_{\mu, \nu, l}^{\gamma, \delta, k} \biggl( \omega^{\prime} \biggl(v-\frac{1}{b} \biggr) ^{\mu} \biggr)\,\mathrm{d}v \\ &\quad \leq \int_{\frac{a+b}{2ab}}^{\frac{1}{a}} \biggl(\frac{1}{a}-u \biggr) ^{\nu-1}E_{\mu, \nu, l}^{\gamma, \delta, k} \biggl( \omega^{\prime} \biggl(\frac{1}{a}-u \biggr) ^{\mu} \biggr) (f\circ h ) (u)\,\mathrm {d}u \\ &\qquad{}+ \int_{\frac{1}{b}}^{\frac{a+b}{2ab}} \biggl(v-\frac{1}{b} \biggr) ^{\nu-1}E_{\mu, \nu, l}^{\gamma, \delta, k} \biggl( \omega^{\prime} \biggl(v-\frac{1}{b} \biggr) ^{\mu} \biggr) (f\circ h ) (v)\,\mathrm {d}v. \end{aligned}$$

This implies

3.11 $$ \begin{aligned}[b] &f \biggl(\frac{2ab}{a+b} \biggr) \bigl( \varepsilon_{\mu, \nu, l, \omega ^{\prime}, (\frac{a+b}{2ab} )^{-}}^{\gamma, \delta, k}1 \bigr) \biggl(\frac{1}{b} \biggr) \\ &\quad \leq\frac{1}{2} \biggl[ \bigl( \varepsilon_{\mu, \nu, l, \omega^{\prime}, (\frac{a+b}{2ab} )^{-}}^{\gamma, \delta, k}f \circ h \bigr) \biggl(\frac{1}{b} \biggr)+ \bigl( \varepsilon_{\mu, \nu, l, \omega^{\prime}, (\frac{a+b}{2ab} )^{+}}^{\gamma, \delta, k}f \circ h \bigr) \biggl(\frac{1}{a} \biggr) \biggr]. \end{aligned} $$

On the other hand, the harmonic convexity of *f* yields

3.12 $$ f \biggl(\frac{ab}{\frac{t}{2}a+\frac{2-t}{2}b} \biggr) +f \biggl(\frac {ab}{\frac{t}{2}b+\frac{2-t}{2}a} \biggr)\leq f(a)+f(b) $$

for all $t\in[0,1]$. Multiplying both sides of (3.12) by $t^{\nu-1}E_{\mu, \nu, l}^{\gamma, \delta, k} ( \omega t^{\mu} )$ and integrating over $[0,1]$, we have

$$\begin{aligned} & \int_{0}^{1}t^{\nu-1}E_{\mu, \nu, l}^{\gamma, \delta, k} \bigl( \omega t^{\mu} \bigr)f \biggl(\frac{ab}{\frac{t}{2}a+\frac{2-t}{2}b} \biggr) \,\mathrm{d}t+ \int_{0}^{1}t^{\nu-1}E_{\mu, \nu, l}^{\gamma, \delta, k} \bigl( \omega t^{\mu} \bigr)f \biggl(\frac{ab}{\frac{t}{2}b+\frac {2-t}{2}a} \biggr) \,\mathrm{d}t \\ &\quad \leq \bigl(f(a)+f(b) \bigr) \int_{0}^{1}t^{\nu-1}E_{\mu, \nu, l}^{\gamma, \delta, k} \bigl( \omega t^{\mu} \bigr)\,\mathrm{d}t . \end{aligned}$$

This implies, using substitutions (3.10),

$$\begin{aligned} & \int_{\frac{a+b}{2ab}}^{\frac{1}{a}} \biggl(\frac{1}{a}-u \biggr) ^{\nu -1}E_{\mu, \nu, l}^{\gamma, \delta, k} \biggl( \omega^{\prime} \biggl(\frac {1}{a}-u \biggr) ^{\mu} \biggr) (f\circ h ) (u) \,\mathrm{d}u \\ &\qquad{}+ \int_{\frac{1}{b}}^{\frac{a+b}{2ab}} \biggl(v-\frac{1}{b} \biggr) ^{\nu -1}E_{\mu, \nu, l}^{\gamma, \delta, k} \biggl( \omega^{\prime} \biggl(v-\frac{1}{b} \biggr) ^{\mu} \biggr) (f\circ h ) (v)\,\mathrm {d}v. \\ &\quad \leq \bigl(f(a)+f(b) \bigr) \int_{\frac{a+b}{2ab}}^{\frac{1}{a}} \biggl(\frac{1}{a}-u \biggr) ^{\nu-1}E_{\mu, \nu, l}^{\gamma, \delta, k} \biggl( \omega^{\prime} \biggl(\frac{1}{a}-u \biggr) ^{\mu} \biggr) (f\circ h ) (u) \,\mathrm{d}u . \end{aligned}$$

So

3.13 $$\begin{aligned} &\frac{1}{2} \biggl[ \bigl( \varepsilon_{\mu, \nu, l, \omega^{\prime}, (\frac{a+b}{2ab} )^{-}}^{\gamma, \delta, k}f \circ h \bigr) \biggl(\frac {1}{b} \biggr)+ \bigl( \varepsilon_{\mu, \nu, l, \omega^{\prime}, (\frac{a+b}{2ab} )^{+}}^{\gamma, \delta, k}f \circ h \bigr) \biggl(\frac {1}{a} \biggr) \biggr] \\ &\quad \leq\frac{f(a)+f(b)}{2} \bigl( \varepsilon_{\mu, \nu, l, \omega^{\prime }, (\frac{a+b}{2ab} )^{+}}^{\gamma, \delta, k}1 \bigr) \biggl(\frac {1}{a} \biggr). \end{aligned}$$

Combining (3.11) and (3.13), we get (3.7). □

### Remark

In Theorem 3.3, if we take $\omega=0$, then we get the known inequality of Işan et al. [7]

$$\begin{aligned} &f \biggl(\frac{2ab}{a+b} \biggr)\leq\frac{\Gamma(\nu+1)}{2^{1-\nu }} \biggl[J_{\frac{a+b}{2ab}+}(f \circ h) \biggl(\frac{1}{a} \biggr)+J_{\frac {a+b}{2ab}-}(f\circ h) \biggl( \frac{1}{b} \biggr) \biggr] \leq\frac {f(a)+f(b)}{2}. \end{aligned}$$

### Theorem 3.4

*Let* $f:I\subset(0, +\infty)\to\mathbb{R}$ *be a harmonically convex functsuch that* $f\in L[a,b]$. *If* $g:[a,b]\to\mathbb{R}$ *is nonnegative*, *integrable*, *and harmonically symmetric with respect to* $\frac {2ab}{a+b}$, *then the following inequality holds*:

3.14 $$\begin{aligned} &f \biggl(\frac{2ab}{a+b} \biggr) \biggl[ \bigl( \varepsilon_{\mu, \nu, l, \omega^{\prime}, (\frac{a+b}{2ab} )^{+}}^{\gamma, \delta, k}g\circ h \bigr) \biggl( \frac{1}{a} \biggr)+ \bigl( \varepsilon_{\mu, \nu , l, \omega^{\prime}, (\frac{a+b}{2ab} )^{-}}^{\gamma, \delta, k}g\circ h \bigr) \biggl( \frac{1}{b} \biggr) \biggr] \\ &\quad \leq\frac{1}{2} \biggl[ \bigl( \varepsilon_{\mu, \nu, l, \omega^{\prime }, (\frac{a+b}{2ab} )^{+}}^{\gamma, \delta, k}fg \circ h \bigr) \biggl( \frac{1}{a} \biggr)+ \bigl( \varepsilon_{\mu, \nu, l, \omega ^{\prime}, (\frac{a+b}{2ab} )^{-}}^{\gamma, \delta, k}fg \circ h \bigr) \biggl( \frac{1}{b} \biggr) \biggr] \\ &\quad \leq\frac{f(a)+f(b)}{2} \biggl[ \bigl( \varepsilon_{\mu, \nu, l, \omega ^{\prime}, (\frac{a+b}{2ab} )^{+}}^{\gamma, \delta, k}g \circ h \bigr) \biggl( \frac{1}{a} \biggr)+ \bigl( \varepsilon_{\mu, \nu, l, \omega^{\prime}, (\frac{a+b}{2ab} )^{-}}^{\gamma, \delta, k}g \circ h \bigr) \biggl( \frac{1}{b} \biggr) \biggr], \end{aligned}$$

*where* $h(x)=\frac{1}{x}$, $x\in [\frac{1}{b}, \frac{1}{a} ] $, *and* $\omega^{\prime}= ( \frac{2ab}{b-a} ) ^{\mu}\omega$.

### Proof

Since *f* ia a harmonically convex function on $[a,b]$, multiplying both sides of (3.9) by $2t^{\nu-1}E_{\mu, \nu, l}^{\gamma, \delta, k} ( \omega t^{\mu} )g (\frac {ab}{\frac{t}{2}a+\frac{2-t}{2}b} )$ and then integrating the resulting inequality over $[0,1]$, we obtain

$$\begin{aligned} &2f \biggl(\frac{2ab}{a+b} \biggr) \int_{0}^{1}t^{\nu-1}E_{\mu, \nu, l}^{\gamma, \delta, k} \bigl( \omega t^{\mu} \bigr)g \biggl(\frac {ab}{\frac{t}{2}a+\frac{2-t}{2}b} \biggr) \,\mathrm{d}t \\ &\quad \leq \int_{0}^{1}t^{\nu-1}E_{\mu, \nu, l}^{\gamma, \delta, k} \bigl( \omega t^{\mu} \bigr)f \biggl(\frac{ab}{\frac{t}{2}a+\frac {2-t}{2}b} \biggr)g \biggl( \frac{ab}{\frac{t}{2}a+\frac{2-t}{2}b} \biggr)\,\mathrm{d}t \\ &\qquad{}+ \int_{0}^{1}t^{\nu-1}E_{\mu, \nu, l}^{\gamma, \delta, k} \bigl( \omega t^{\mu} \bigr)f \biggl(\frac{ab}{\frac{t}{2}b+\frac {2-t}{2}a} \biggr)g \biggl( \frac{ab}{\frac{t}{2}a+\frac{2-t}{2}b} \biggr)\,\mathrm{d}t . \end{aligned}$$

Since *g* is harmonically symmetric with respect to $\frac{2ab}{a+b}$, using Definition 2.2, we have $g ( \frac{1}{x} )=g (\frac{1}{\frac{1}{a}+\frac{1}{b}-x} ) $ for all $x\in [\frac{1}{b}, \frac{1}{a} ] $. Setting $x=\frac{1}{ab} (\frac{t}{2}a+\frac{2-t}{2}b ) $ gives

$$\begin{aligned} &2f \biggl(\frac{2ab}{a+b} \biggr) \int_{\frac{a+b}{2ab}}^{\frac {1}{a}} \biggl(\frac{1}{a}-x \biggr) ^{\nu-1}E_{\mu, \nu, l}^{\gamma, \delta, k} \biggl( \omega^{\prime} \biggl(\frac{1}{a}-x \biggr)^{\mu} \biggr)g \biggl( \frac{1}{x} \biggr)\,\mathrm{d}x \\ &\quad \leq\frac{1}{2} \biggl[ \int_{\frac{a+b}{2ab}}^{\frac{1}{a}} \biggl(\frac {1}{a}-x \biggr)^{\nu-1}E_{\mu, \nu, l}^{\gamma, \delta, k} \biggl( \omega ^{\prime} \biggl(\frac{1}{a}-x \biggr)^{\mu} \biggr)f \biggl( \frac{1}{x} \biggr)g \biggl(\frac{1}{x} \biggr)\,\mathrm{d}x \\ &\qquad{}+ \int_{\frac{a+b}{2ab}}^{\frac{1}{a}} \biggl(\frac {1}{a}-x \biggr)^{\nu-1}E_{\mu, \nu, l}^{\gamma, \delta, k} \biggl( \omega ^{\prime} \biggl(\frac{1}{a}-x \biggr)^{\mu} \biggr)f \biggl( \frac{1}{\frac {1}{a}+\frac{1}{b}-x} \biggr)g \biggl(\frac{1}{x} \biggr)\,\mathrm {d}x \biggr] . \end{aligned}$$

Using substitution $u=\frac{1}{a}+\frac{1}{b}-x$, we have

$$\begin{aligned} &2f \biggl(\frac{2ab}{a+b} \biggr) \int_{\frac{a+b}{2ab}}^{\frac {1}{a}} \biggl(\frac{1}{a}-x \biggr) ^{\nu-1}E_{\mu, \nu, l}^{\gamma, \delta, k} \biggl( \omega^{\prime} \biggl(\frac{1}{a}-x \biggr)^{\mu} \biggr)g \biggl( \frac{1}{x} \biggr)\,\mathrm{d}x \\ &\quad \leq\frac{1}{2} \biggl[ \int_{\frac{a+b}{2ab}}^{\frac{1}{a}} \biggl(\frac {1}{a}-x \biggr)^{\nu-1}E_{\mu, \nu, l}^{\gamma, \delta, k} \biggl( \omega ^{\prime} \biggl(\frac{1}{a}-x \biggr)^{\mu} \biggr)f \biggl( \frac{1}{x} \biggr)g \biggl(\frac{1}{x} \biggr)\,\mathrm{d}x \\ &\qquad{}+ \int_{\frac{1}{b}}^{\frac{a+b}{2ab}} \biggl(u-\frac {1}{b} \biggr)^{\nu-1}E_{\mu, \nu, l}^{\gamma, \delta, k} \biggl( \omega ^{\prime} \biggl(u-\frac{1}{b} \biggr)^{\mu} \biggr)f \biggl( \frac{1}{u} \biggr)g \biggl(\frac{1}{u} \biggr)\,\mathrm{d}x \biggr]. \end{aligned}$$

Hence, using Lemma 3.1, we obtain

3.15 $$\begin{aligned} &f \biggl(\frac{2ab}{a+b} \biggr) \biggl[ \bigl( \varepsilon_{\mu, \nu, l, \omega, (\frac{a+b}{2ab} )^{+}}^{\gamma, \delta, k}g\circ h \bigr) \biggl( \frac{1}{a} \biggr)+ \bigl( \varepsilon_{\mu, \nu, l, \omega, (\frac{a+b}{2ab} )^{-}}^{\gamma, \delta, k}g\circ h \bigr) \biggl( \frac{1}{b} \biggr) \biggr] \\ &\quad \leq\frac{1}{2} \biggl[ \bigl( \varepsilon_{\mu, \nu, l, \omega^{\prime }, (\frac{a+b}{2ab} )^{+}}^{\gamma, \delta, k}fg \circ h \bigr) \biggl( \frac{1}{a} \biggr)+ \bigl( \varepsilon_{\mu, \nu, l, \omega ^{\prime}, (\frac{a+b}{2ab} )^{-}}^{\gamma, \delta, k}fg \circ h \bigr) \biggl( \frac{1}{b} \biggr) \biggr]. \end{aligned}$$

For the proof of the second inequality in (3.14), we first note that *f* is a harmonically convex function. Then, multiplying both sides of (3.12) by $2t^{\nu-1}E_{\mu, \nu, l}^{\gamma, \delta, k} ( \omega t^{\mu} )g (\frac{ab}{\frac{t}{2}a+\frac {2-t}{2}b} )$ and integrating the resulting inequality over $[0,1]$, we obtain

$$\begin{aligned} & \int_{0}^{1}t^{\nu-1}E_{\mu, \nu, l}^{\gamma, \delta, k} \bigl( \omega t^{\mu} \bigr)f \biggl(\frac{ab}{\frac{t}{2}a+\frac{2-t}{2}b} \biggr)g \biggl( \frac{ab}{\frac{t}{2}a+\frac{2-t}{2}b} \biggr)\,\mathrm{d}t \\ &\qquad{}+ \int_{0}^{1}t^{\nu-1}E_{\mu, \nu, l}^{\gamma, \delta, k} \bigl( \omega t^{\mu} \bigr)f \biggl(\frac{ab}{\frac{t}{2}b+\frac{2-t}{2}a} \biggr)g \biggl( \frac{ab}{\frac{t}{2}a+\frac{2-t}{2}b} \biggr)\,\mathrm{d}t \\ &\quad \leq \bigl(f(a)+f(b) \bigr) \int_{0}^{1}t^{\nu-1}E_{\mu, \nu, l}^{\gamma , \delta, k} \bigl( \omega t^{\mu} \bigr)g \biggl(\frac{ab}{\frac {t}{2}a+\frac{2-t}{2}b} \biggr) \,\mathrm{d}t. \end{aligned} $$

From this, using Lemma 3.1, we get

3.16 $$ \begin{aligned} &\frac{1}{2} \biggl[ \bigl( \varepsilon_{\mu, \nu, l, \omega^{\prime}, (\frac{a+b}{2ab} )^{+}}^{\gamma, \delta, k}fg \circ h \bigr) \biggl( \frac{1}{a} \biggr)+ \bigl( \varepsilon_{\mu, \nu, l, \omega^{\prime}, (\frac{a+b}{2ab} )^{-}}^{\gamma, \delta, k}fg \circ h \bigr) \biggl( \frac{1}{b} \biggr) \biggr] \\ &\quad \leq\frac{f(a)+f(b)}{2} \biggl[ \bigl( \varepsilon_{\mu, \nu, l, \omega ^{\prime}, (\frac{a+b}{2ab} )^{+}}^{\gamma, \delta, k}g \circ h \bigr) \biggl( \frac{1}{a} \biggr)+ \bigl( \varepsilon_{\mu, \nu, l, \omega^{\prime}, (\frac{a+b}{2ab} )^{-}}^{\gamma, \delta, k}g \circ h \bigr) \biggl( \frac{1}{b} \biggr) \biggr]. \end{aligned} $$

From (3.15) and (3.16) we obtain (3.14). The proof is completed. □

### Theorem 3.5

*Let* $f:I\subset(0, +\infty)\to\mathbb{R}$ *be a differentiable function such that* $f^{\prime}\in L[a,b]$, *where* $a,b\in I$, $a< b$. *If* $\vert f^{\prime} \vert $ *is a harmonically convex function and* $g:[a,b]\to\mathbb{R}$ *is continuous and harmonically symmetric with respect to* $\frac{2ab}{a+b}$, *then the following inequality holds*:

3.17 $$\begin{aligned} & \biggl\vert f \biggl(\frac{2ab}{a+b} \biggr) \biggl[ \bigl( \varepsilon_{\mu, \nu, l, \omega^{\prime}, (\frac{a+b}{2ab} )^{+}}^{\gamma, \delta, k}g\circ h \bigr) \biggl( \frac{1}{a} \biggr)+ \bigl( \varepsilon_{\mu, \nu, l, \omega^{\prime}, (\frac{a+b}{2ab} )^{-}}^{\gamma, \delta , k}g\circ h \bigr) \biggl( \frac{1}{b} \biggr) \biggr] \\ &\qquad{}- \biggl[ \bigl( \varepsilon_{\mu, \nu, l, \omega^{\prime }, (\frac{a+b}{2ab} )^{+}}^{\gamma, \delta, k}fg\circ h \bigr) \biggl( \frac{1}{a} \biggr)+ \bigl( \varepsilon_{\mu, \nu, l, \omega ^{\prime}, (\frac{a+b}{2ab} )^{-}}^{\gamma, \delta, k}fg \circ h \bigr) \biggl( \frac{1}{b} \biggr) \biggr] \biggr\vert \\ &\quad \leq \biggl\vert f \biggl(\frac{2ab}{a+b} \biggr) \biggr\vert \Vert g \Vert _{\infty} \biggl[ \bigl( \varepsilon_{\mu, \nu, l, \omega ^{\prime}, (\frac{a+b}{2ab} )^{+}}^{\gamma, \delta, k}1 \bigr) \biggl( \frac{1}{a} \biggr)+ \bigl( \varepsilon_{\mu, \nu, l, \omega ^{\prime}, (\frac{a+b}{2ab} )^{-}}^{\gamma, \delta, k}1 \bigr) \biggl( \frac{1}{b} \biggr) \biggr] \\ &\qquad{}+S \Vert g \Vert _{\infty} \biggl( \frac{b-a}{2ab} \biggr) ^{\nu}\cdot\frac{\nu+4}{4\nu (\nu+1 ) } \bigl( \bigl\vert f(a) \bigr\vert + \bigl\vert f(b) \bigr\vert \bigr), \end{aligned}$$

*where* $h(x)=\frac{1}{x}$, $x\in [\frac{1}{b}, \frac{1}{a} ] $, $\omega^{\prime}= ( \frac{2ab}{b-a} ) ^{\mu}\omega$, *and* $\vert E_{\mu, \nu, l}^{\gamma, \delta, k} ( t ) \vert \leq S$.

### Proof

From Lemma 3.2, relationships (3.3) and (3.5), and the property of the modulus we have

$$\begin{aligned} J&= \biggl\vert f \biggl(\frac{2ab}{a+b} \biggr) \biggl[ \bigl( \varepsilon_{\mu, \nu, l, \omega^{\prime}, (\frac{a+b}{2ab} )^{+}}^{\gamma, \delta, k}g\circ h \bigr) \biggl( \frac{1}{a} \biggr)+ \bigl( \varepsilon_{\mu, \nu, l, \omega^{\prime}, (\frac{a+b}{2ab} )^{-}}^{\gamma, \delta , k}g\circ h \bigr) \biggl( \frac{1}{b} \biggr) \biggr] \\ &\quad{}- \biggl[ \bigl( \varepsilon_{\mu, \nu, l, \omega^{\prime }, (\frac{a+b}{2ab} )^{+}}^{\gamma, \delta, k}fg\circ h \bigr) \biggl( \frac{1}{a} \biggr)+ \bigl( \varepsilon_{\mu, \nu, l, \omega ^{\prime}, (\frac{a+b}{2ab} )^{-}}^{\gamma, \delta, k}fg \circ h \bigr) \biggl( \frac{1}{b} \biggr) \biggr] \biggr\vert \\ &\leq \biggl\vert f \biggl(\frac{2ab}{a+b} \biggr) \biggr\vert \int_{\frac {1}{b}}^{\frac{2ab}{a+b}} \biggl(s-\frac{1}{b} \biggr) ^{\nu-1} \biggl\vert E_{\mu, \nu, l}^{\gamma, \delta, k} \biggl( \omega^{\prime} \biggl(s- \frac {1}{b} \biggr) ^{\mu} \biggr) \biggr\vert \bigl\vert (g\circ h ) (s) \bigr\vert \,\mathrm{d}s \\ &\quad{}+ \int_{\frac{1}{b}}^{\frac{a+b}{2ab}} \biggl(t-\frac{1}{b} \biggr) ^{\nu -1} \biggl\vert E_{\mu, \nu, l}^{\gamma, \delta, k} \biggl( \omega^{\prime } \biggl(t- \frac{1}{b} \biggr) ^{\mu} \biggr) \biggr\vert \bigl\vert (fg\circ h ) (t) \bigr\vert \,\mathrm{d}t \\ &\begin{aligned} &\quad{}+ \biggl\vert f \biggl(\frac{2ab}{a+b} \biggr) \biggr\vert \int_{\frac {a+b}{2ab}}^{\frac{1}{a}} \biggl(\frac{1}{a}-s \biggr) ^{\nu-1} \biggl\vert E_{\mu, \nu, l}^{\gamma, \delta, k} \biggl( \omega^{\prime} \biggl(\frac{1}{a}-s \biggr) ^{\mu} \biggr) \biggr\vert \bigl\vert (g\circ h ) (s) \bigr\vert \,\mathrm{d}s \\ &\quad{}+ \int_{\frac{a+b}{2ab}}^{\frac{1}{a}} \biggl(\frac{1}{a}-t \biggr) ^{\nu -1} \biggl\vert E_{\mu, \nu, l}^{\gamma, \delta, k} \biggl( \omega^{\prime } \biggl(\frac{1}{a}-t \biggr) ^{\mu} \biggr) \biggr\vert \bigl\vert (fg\circ h ) (t) \bigr\vert \,\mathrm{d}t. \end{aligned} \end{aligned}$$

Since $\Vert g \Vert _{\infty}=\sup_{t\in[a,b]} \vert g(t) \vert $ and $\vert E_{\mu, \nu, l}^{\gamma, \delta, k} ( t ) \vert \leq S$, we have

3.18 $$\begin{aligned} J&\leq \biggl\vert f \biggl(\frac{2ab}{a+b} \biggr) \biggr\vert \Vert g \Vert _{\infty} \int_{\frac{1}{b}}^{\frac{2ab}{a+b}} \biggl(s-\frac {1}{b} \biggr) ^{\nu-1} E_{\mu, \nu, l}^{\gamma, \delta, k} \biggl( \omega ^{\prime} \biggl(s- \frac{1}{b} \biggr) ^{\mu} \biggr)\,\mathrm{d}s \\ &\quad{}+S \Vert g \Vert _{\infty} \int_{\frac{1}{b}}^{\frac {a+b}{2ab}} \biggl(t-\frac{1}{b} \biggr) ^{\nu-1} \bigl\vert (f\circ h ) (t) \bigr\vert \,\mathrm{d}t \\ &\quad{}+ \biggl\vert f \biggl(\frac{2ab}{a+b} \biggr) \biggr\vert \Vert g \Vert _{\infty} \int_{\frac{a+b}{2ab}}^{\frac{1}{a}} \biggl(\frac {1}{a}-s \biggr) ^{\nu-1} E_{\mu, \nu, l}^{\gamma, \delta, k} \biggl( \omega^{\prime} \biggl(\frac{1}{a}-s \biggr) ^{\mu} \biggr)\,\mathrm{d}s \\ &\quad{}+S \Vert g \Vert _{\infty} \int_{\frac{a+b}{2ab}}^{\frac {1}{a}} \biggl(\frac{1}{a}-t \biggr) ^{\nu-1} \bigl\vert (f\circ h ) (t) \bigr\vert \,\mathrm{d}t \\ &= \biggl\vert f \biggl(\frac{2ab}{a+b} \biggr) \biggr\vert \Vert g \Vert _{\infty} \biggl[ \bigl( \varepsilon_{\mu, \nu, l, \omega^{\prime}, (\frac{a+b}{2ab} )^{+}}^{\gamma, \delta, k}g \circ h \bigr) \biggl( \frac{1}{a} \biggr)+ \bigl( \varepsilon_{\mu, \nu, l, \omega^{\prime}, (\frac{a+b}{2ab} )^{-}}^{\gamma, \delta, k}g \circ h \bigr) \biggl( \frac{1}{b} \biggr) \biggr] \\ &\quad{}+S \Vert g \Vert _{\infty} (J_{1}+J_{2} ). \end{aligned}$$

Setting $t=\frac{1}{ab} (\frac{u}{2}a+\frac{2-u}{2}b ) $ and using the harmonic convexity of $\vert f \vert $, we have

$$\begin{aligned} J_{1}&= \int_{\frac{1}{b}}^{\frac{a+b}{2ab}} \biggl(t-\frac{1}{b} \biggr) ^{\nu-1} \bigl\vert (f\circ h ) (t) \bigr\vert \,\mathrm{d}t= \int _{\frac{1}{b}}^{\frac{a+b}{2ab}} \biggl(t-\frac{1}{b} \biggr) ^{\nu -1} \biggl\vert f \biggl(\frac{1}{t} \biggr) \biggr\vert \,\mathrm{d}t \\ &=\frac{1}{2} \biggl( \frac{b-a}{ab} \biggr) ^{\nu} \int_{1}^{2} \biggl(1-\frac{1}{2}u \biggr) ^{\nu-1} \biggl\vert f \biggl(\frac{ab}{\frac {u}{2}a+\frac{2-u}{2}b} \biggr) \biggr\vert \,\mathrm{d}u \\ &\leq\frac{1}{2} \biggl( \frac{b-a}{ab} \biggr) ^{\nu} \int_{1}^{2} \biggl(1-\frac{1}{2}u \biggr) ^{\nu-1} \biggl( \frac{u}{2} \bigl\vert f(b) \bigr\vert + \frac{2-u}{2} \bigl\vert f(a) \bigr\vert \biggr)\,\mathrm{d}u. \end{aligned}$$

This implies

3.19 $$ J_{1}\leq\frac{1}{4} \biggl( \frac{b-a}{ab} \biggr) ^{\nu} \biggl(\frac {2\nu+4}{\nu(\nu+1)} \bigl\vert f(b) \bigr\vert -\frac{1}{\nu+1} \bigl\vert f(a) \bigr\vert \biggr). $$

Similarly, using the substitution $t=\frac{1}{ab} (\frac {v}{2}b+\frac{2-v}{2}a ) $, we get

3.20 $$\begin{aligned} J_{2}&= \int_{\frac{a+b}{2ab}}^{\frac{1}{a}} \biggl(\frac{1}{a}-t \biggr) ^{\nu-1} \bigl\vert (f\circ h ) (t) \bigr\vert \,\mathrm{d}t= \int _{\frac{a+b}{2ab}}^{\frac{1}{a}} \biggl(\frac{1}{a}-t \biggr) ^{\nu -1} \biggl\vert f \biggl(\frac{1}{t} \biggr) \biggr\vert \,\mathrm{d}t \\ &\leq\frac{1}{4} \biggl( \frac{b-a}{ab} \biggr) ^{\nu} \biggl(\frac{2\nu +4}{\nu(\nu+1)} \bigl\vert f(a) \bigr\vert -\frac{1}{\nu+1} \bigl\vert f(b) \bigr\vert \biggr). \end{aligned}$$

Substituting (3.19) and (3.20) into (3.18), we obtain (3.17). The proof is completed. □

### Theorem 3.6

*Let* $f:I\subset(0, +\infty)\to\mathbb{R}$ *be a differentiable function on* $I^{\circ}$, *the interior of* *I*, *such that* $f^{\vert}\in L[a,b]$, *where* $a,b\in I$. *If* $\vert f \vert ^{q}$, $q\geq1$, *is a harmonically convex function on* $[a,b]$, $g:[a,b]\to\mathbb{R}$ *is continuous and harmonically symmetric with respect to* $\frac {2ab}{a+b}$, *then the following inequality holds*:

3.21 $$\begin{aligned} & \biggl\vert f \biggl(\frac{2ab}{a+b} \biggr) \biggl[ \bigl( \varepsilon_{\mu, \nu, l, \omega^{\prime}, (\frac{a+b}{2ab} )^{+}}^{\gamma, \delta, k}g\circ h \bigr) \biggl( \frac{1}{a} \biggr)+ \bigl( \varepsilon_{\mu, \nu, l, \omega^{\prime}, (\frac{a+b}{2ab} )^{-}}^{\gamma, \delta , k}g\circ h \bigr) \biggl( \frac{1}{b} \biggr) \biggr] \\ &\qquad{}- \biggl[ \bigl( \varepsilon_{\mu, \nu, l, \omega^{\prime }, (\frac{a+b}{2ab} )^{+}}^{\gamma, \delta, k}fg\circ h \bigr) \biggl( \frac{1}{a} \biggr)+ \bigl( \varepsilon_{\mu, \nu, l, \omega ^{\prime}, (\frac{a+b}{2ab} )^{-}}^{\gamma, \delta, k}fg \circ h \bigr) \biggl( \frac{1}{b} \biggr) \biggr] \biggr\vert \\ &\quad \leq \biggl\vert f \biggl(\frac{2ab}{a+b} \biggr) \biggr\vert \Vert g \Vert _{\infty} \biggl[ \bigl( \varepsilon_{\mu, \nu, l, \omega ^{\prime}, (\frac{a+b}{2ab} )^{+}}^{\gamma, \delta, k}1 \bigr) \biggl( \frac{1}{a} \biggr)+ \bigl( \varepsilon_{\mu, \nu, l, \omega ^{\prime}, (\frac{a+b}{2ab} )^{-}}^{\gamma, \delta, k}1 \bigr) \biggl( \frac{1}{b} \biggr) \biggr] \\ &\qquad{}+S \Vert g \Vert _{\infty} \biggl( \frac{1}{\nu} \biggr) ^{1-1/q} \biggl( \frac{1}{\nu+1 } \biggr) ^{1/q} \biggl( \frac{1}{2} \biggr) ^{\nu+1/q} \\ &\qquad{}\times \biggl[ \biggl( \frac{1}{\nu} \bigl\vert f(b) \bigr\vert ^{q} + \bigl\vert f(a) \bigr\vert ^{q} \biggr)^{1/q}+ \biggl(\frac{1}{\nu} \bigl\vert f(a) \bigr\vert ^{q} + \bigl\vert f(b) \bigr\vert ^{q} \biggr)^{1/q} \biggr] , \end{aligned}$$

*where* $h(x)=\frac{1}{x}$, $x\in [\frac{1}{b}, \frac{1}{a} ] $, $\omega^{\prime}= ( \frac{2ab}{b-a} ) ^{\mu}\omega$, *and* $\vert E_{\mu, \nu, l}^{\gamma, \delta, k} ( t ) \vert \leq S$.

### Proof

From (3.18) we have

$$\begin{aligned} J&\leq \biggl\vert f \biggl(\frac{2ab}{a+b} \biggr) \biggr\vert \Vert g \Vert _{\infty} \int_{\frac{1}{b}}^{\frac{2ab}{a+b}} \biggl(s-\frac {1}{b} \biggr) ^{\nu-1} E_{\mu, \nu, l}^{\gamma, \delta, k} \biggl( \omega ^{\prime} \biggl(s- \frac{1}{b} \biggr) ^{\mu} \biggr)\,\mathrm{d}s \\ &\quad{}+S \Vert g \Vert _{\infty} \int_{\frac{1}{b}}^{\frac {a+b}{2ab}} \biggl(t-\frac{1}{b} \biggr) ^{\nu-1} \bigl\vert (f\circ h ) (t) \bigr\vert \,\mathrm{d}t \\ &\quad{}+ \biggl\vert f \biggl(\frac{2ab}{a+b} \biggr) \biggr\vert \Vert g \Vert _{\infty} \int_{\frac{a+b}{2ab}}^{\frac{1}{a}} \biggl(\frac {1}{a}-s \biggr) ^{\nu-1} E_{\mu, \nu, l}^{\gamma, \delta, k} \biggl( \omega^{\prime} \biggl(\frac{1}{a}-s \biggr) ^{\mu} \biggr)\,\mathrm{d}s \\ &\quad{}+S \Vert g \Vert _{\infty} \int_{\frac{a+b}{2ab}}^{\frac {1}{a}} \biggl(\frac{1}{a}-t \biggr) ^{\nu-1} \bigl\vert (f\circ h ) (t) \bigr\vert \,\mathrm{d}t \\ &= \biggl\vert f \biggl(\frac{2ab}{a+b} \biggr) \biggr\vert \Vert g \Vert _{\infty} \biggl[ \bigl( \varepsilon_{\mu, \nu, l, \omega^{\prime}, (\frac{a+b}{2ab} )^{+}}^{\gamma, \delta, k}g \circ h \bigr) \biggl( \frac{1}{a} \biggr)+ \bigl( \varepsilon_{\mu, \nu, l, \omega^{\prime}, (\frac{a+b}{2ab} )^{-}}^{\gamma, \delta, k}g \circ h \bigr) \biggl( \frac{1}{b} \biggr) \biggr] \\ &\quad{}+S \Vert g \Vert _{\infty} \biggl[ \int_{\frac{1}{b}}^{\frac {a+b}{2ab}} \biggl(t-\frac{1}{b} \biggr) ^{\nu-1} \biggl\vert f \biggl(\frac {1}{t} \biggr) \biggr\vert \,\mathrm{d}t+ \int_{\frac{a+b}{2ab}}^{\frac {1}{a}} \biggl(\frac{1}{a}-t \biggr) ^{\nu-1} \biggl\vert f \biggl(\frac {1}{t} \biggr) \biggr\vert \,\mathrm{d}t \biggr] . \end{aligned}$$

Using the substitutions $t=\frac{1}{ab} (\frac{u}{2}a+\frac {2-u}{2}b )$ and $t=\frac{1}{ab} (\frac{v}{2}b+\frac {2-v}{2}a ) $, the power mean inequality, and the harmonicity of $\vert f \vert ^{q}$, it follows that

$$\begin{aligned} J&\leq \biggl\vert f \biggl(\frac{2ab}{a+b} \biggr) \biggr\vert \Vert g \Vert _{\infty} \biggl[ \bigl( \varepsilon_{\mu, \nu, l, \omega ^{\prime}, (\frac{a+b}{2ab} )^{+}}^{\gamma, \delta, k}g \circ h \bigr) \biggl( \frac{1}{a} \biggr)+ \bigl( \varepsilon_{\mu, \nu, l, \omega^{\prime}, (\frac{a+b}{2ab} )^{-}}^{\gamma, \delta, k}g \circ h \bigr) \biggl( \frac{1}{b} \biggr) \biggr] \\ &\quad{}+\frac{S \Vert g \Vert _{\infty}}{2} \biggl(\frac {b-a}{ab} \biggr) ^{\nu} \\ &\quad{}\times \biggl[ \int_{1}^{2} \biggl(1-\frac{1}{2}u \biggr) ^{\nu -1} \biggl\vert f \biggl(\frac{ab}{\frac{u}{2}a+\frac{2-u}{2}b } \biggr) \biggr\vert \,\mathrm{d}u+ \int_{1}^{2} \biggl(1-\frac{1}{2}v \biggr) ^{\nu -1} \biggl\vert f \biggl(\frac{ab}{\frac{v}{2}b+\frac{2-v}{2}a} \biggr) \biggr\vert \,\mathrm{d}v \biggr] \\ &\leq \biggl\vert f \biggl(\frac{2ab}{a+b} \biggr) \biggr\vert \Vert g \Vert _{\infty} \biggl[ \bigl( \varepsilon_{\mu, \nu, l, \omega ^{\prime}, (\frac{a+b}{2ab} )^{+}}^{\gamma, \delta, k}g \circ h \bigr) \biggl( \frac{1}{a} \biggr)+ \bigl( \varepsilon_{\mu, \nu, l, \omega^{\prime}, (\frac{a+b}{2ab} )^{-}}^{\gamma, \delta, k}g \circ h \bigr) \biggl( \frac{1}{b} \biggr) \biggr] \\ &\quad{}+\frac{S \Vert g \Vert _{\infty}}{2} \biggl(\frac {b-a}{ab} \biggr) ^{\nu} \biggl[ \biggl( \int_{1}^{2} \biggl(1-\frac{1}{2}u \biggr) ^{\nu -1}\,\mathrm{d}u \biggr) ^{1-1/q} \\ &\quad{}\times \biggl( \int_{1}^{2} \biggl(1-\frac{1}{2}u \biggr) ^{\nu-1} \biggl( \frac{u}{2} \bigl\vert f(b) \bigr\vert ^{q}+\frac {2-u}{2} \bigl\vert f(a) \bigr\vert ^{q} \biggr)\,\mathrm{d}u \biggr) ^{1/q} \\ &\quad{}+ \biggl( \int_{1}^{2} \biggl(1-\frac{1}{2}v \biggr) ^{\nu -1}\,\mathrm{d}v \biggr) ^{1-1/q} \biggl( \int_{1}^{2} \biggl(1-\frac{1}{2}v \biggr) ^{\nu-1} \biggl( \frac{v}{2} \bigl\vert f(a) \bigr\vert ^{q}+\frac {2-v}{2} \bigl\vert f(b) \bigr\vert ^{q} \biggr)\,\mathrm{d}v \biggr) ^{1/q} \biggr]. \end{aligned}$$

Making the substitutions $x=1-\frac{1}{2}u$ and $y=1-\frac{1}{2}v $ and simple calculations, we get inequality (3.21). □

## Conclusions

We have obtained several new refinements of Hermite-Hadamard-type inequalities via harmonic convex functions. These results involve particularly the generalized Mittag-Leffler function. We also discussed several particular cases. We expect that the results obtained in this paper may stimulate further research in the field. We would like to specify here that the generalized fractional integral operators containing the Mittag-Leffler function generalize the integrals of Riemann-Liouville type, and the results obtained in this paper can be developed via other different types of convexities.

## References

[1] Cristescu G, Lupsa L (2002). Non-connected Convexities and Applications.

[2] Cristescu G, Noor MA, Awan MU (2015). Bounds of the second degree cumulative frontier gaps of functions with generalized convexity. Carpath. J. Math..

[3] Işcan I (2014). Hermite-Hadamard type inequalities for harmonically convex functions. Hacet. J. Math. Stat..

[4] Noor MA, Noor KI, Awan MU, Costache S (2015). Some integral inequalities for harmonically *h*-convex functions. UPB Sci. Bull., Ser. A.

[5] Işcan I (2014). Hermite-Hadamard and Simpson-like type inequalities for differentiable harmonically convex functions. J. Math..

[6] Işcan I, Wu S (2014). Hermite-Hadamard type inequalities for harmonically convex functions via fractional integrals. Appl. Math. Comput..

[7] Kunt M, Işcan I, Yazici N, Gözütok U (2016). On New Inequalities of Hermite-Hadamard-Fejer Type for Harmonically Convex Functions via Fractional Integrals.

[8] Latif, MA, Dragomir, SS, Momoniat, E: Some Fejer type inequalities for harmonically convex functions with applications to special means. http://rgmia.org/papers/v18/v18a24

[9] Mihai MV, Noor MA, Noor KI, Awan MU (2015). Some integral inequalities for harmonic *h*-convex functions involving hypergeometric functions. Appl. Math. Comput..

[10] Dragomir SS, Pearce CEM (2000). Selected Topics on Hermite-Hadamard Inequalities and Applications.

[11] Set E, Noor MA, Awan MU, Gözpinar A (2017). Generalized Hermite-Hadamard type inequalities for convex functions involving fractional integral operators. J. Inequal. Appl..

[12] Sarikaya MZ, Set E, Yaldiz H, Başak N (2013). Hermite-Hadamard’s inequalities for fractional integrals and related fractional inequalities. Math. Comput. Model..

[13] Mihai MV (2013). Some Hermite-Hadamard type inequalities obtained via Riemann-Liouville fractional calculus. Tamkang J. Math..

[14] Mihai MV (2014). New inequalities for co-ordinated convex functions via Riemann-Liouville fractional calculus. Tamkang J. Math..

[15] Mihai MV, Mitroi FC (2014). Hermite-Hadamard type inequalities obtained via Riemann-Liouville fractional calculus. Acta Math. Univ. Comen..

[16] Awan MU, Noor MA, Mihai MV, Noor KI (2016). Fractional Hermite-Hadamard inequalities for differentiable *s*-Godunova-Levin functions. Filomat.

[17] Sarikaya MZ, Yildirim H (2016). On Hermite-Hadamard type inequalities for Riemann-Liouville fractional integrals. Miskolc Math. Notes.

[18] Nisan KS, Rahman G, Băleanu D, Mubeen S, Arshad M (2017). The $(k,s)$-fractional calculus of *k*-Mittagâ-Leffler function. Adv. Differ. Equ..

[19] Gorenflo R, Mainardi F (1997). Fractional Calculus: Integral and Differential Equations of Fractional Oder.

[20] Kilbas AA, Srivastava HM, Trujillo JJ (2006). Theory and Applications of Fractional Differential Equations.

[21] Salim TO, Faraj AW (2012). A generalization of Mittag-Leffler function and integral operator associated with fractional calculus. J. Fract. Calc. Appl..

[22] Srivastava HM, Tomovski Z̆ (2009). Fractional calculus with an integral operator containing generalized mittagâ-Leffler function in the kernel. Appl. Math. Comput..

[23] Prabhakar TR (1971). A singular integral equation with a generalized mittagâ-Leffler function in the kernel. Yokohama Math. J..

